# Research on the fate of polymeric nanoparticles in the process of the intestinal absorption based on model nanoparticles with various characteristics: size, surface charge and pro-hydrophobics

**DOI:** 10.1186/s12951-021-00770-2

**Published:** 2021-01-27

**Authors:** Shiqi Guo, Yanzi Liang, Lanze Liu, Miaomiao Yin, Aiping Wang, Kaoxiang Sun, Youxin Li, Yanan Shi

**Affiliations:** 1 School of Pharmacy, Key Laboratory of Molecular Pharmacology and Drug Evaluation (Yantai University), Ministry of Education, Collaborative Innovation Center of Advanced Drug Delivery System and Biotech Drugs in Universities of Shandong, Yantai University, Yantai, 264005 People’s Republic of China; 2 College of Life Science, Yantai University, Yantai, 264005 People’s Republic of China; 3 China Resources Double-crane Pharmaceutical Co., Ltd., Beijing, China; 4 State Key Laboratory of Long-acting and Targeting Drug Delivery System, Luye Pharmaceutical Co., Ltd., Yantai, China

**Keywords:** Polymeric nanoparticles, Basic properties, Mucus layer, Intestinal epithelium, Physiological barrier

## Abstract

**Background:**

The use of drug nanocarriers to encapsulate drugs for oral administration may become an important strategy in addressing the challenging oral absorption of some drugs. In this study—with the premise of controlling single variables—we prepared model nanoparticles with different particle sizes, surface charges, and surface hydrophobicity/hydrophilicity. The two key stages of intestinal nanoparticles (NPs) absorption—the intestinal mucus layer penetration stage and the trans-intestinal epithelial cell stage—were decoupled and analyzed. The intestinal absorption of each group of model NPs was then investigated.

**Results:**

Differences in the behavioral trends of NPs in each stage of intestinal absorption were found to result from differences in particle properties. Small size, low-magnitude negative charge, and moderate hydrophilicity helped NPs pass through the small intestinal mucus layer more easily. Once through the mucus layer, an appropriate size, positive surface charge, and hydrophobic properties helped NPs complete the process of transintestinal epithelial cell transport.

**Conclusions:**

To achieve high drug bioavailability, the basic properties of the delivery system must be suitable for overcoming the physiological barrier of the gastrointestinal tract. 
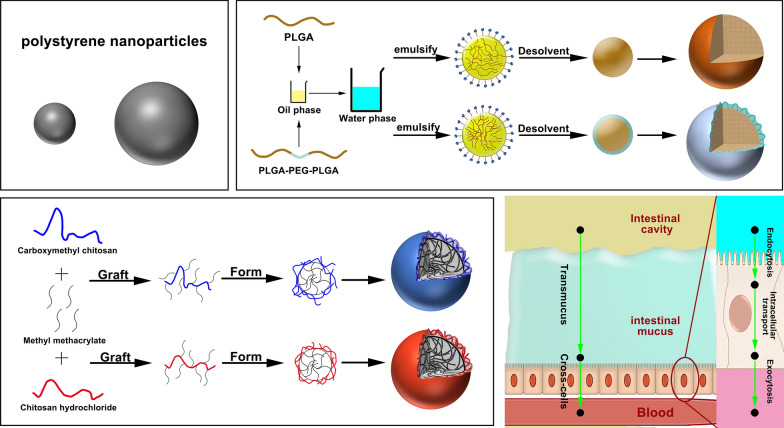

## Background

Nanoscale drug preparations have emerged over the last three decades, and the spread and development of this area since its inception has been rapid. In the field of pharmaceutics nano preparation has become a hot topic. Various nano preparations, including polymer nanoparticles, have been used in drug delivery research to improve bioavailability, solubility, and drug retention time, as well as to reduce drug side effects, such as toxicity. The preparation methods for various nano preparations are becoming increasingly mature, and with the gradual diversification of nanocarrier forms, more complex structures and incidental functions have emerged [1–3]. In terms of application, extensive clinical studies have been conducted on nanoparticle delivery systems, and many particle-based formulations and technologies have been introduced into the clinic. Oral, local, topical, and systemic (such as intravenous) administration are all approved by the US Food and Drug Administration (FDA) for delivery of nanoparticles/microparticles [4]. In the past 3 years, three new nano pharmaceutical preparations (Vyxeos, Onpattro, and Hensify) have been approved and represent a new generation of nano pharmaceutical preparations has successfully entered the market and opened up new clinical trials based on their unique physical and chemical properties. More than 50 nano pharmaceutical preparations have been approved for clinical use, and more than 400 are expected to become new clinical solutions alone or in combination with other key enabling technologies [5–9].

Oral administration is one of the best options for drug administration owing to its non-invasiveness. The oral route has the advantages of avoiding the pain and discomfort caused by injection, as well as eliminating pollution. The human digestive tract, which has a very high surface area and is primarily responsible for the absorption of nutrients, provides an attractive route for orally ingested particles to enter the system. Many oral delivery systems based on polymer nanoparticles have been developed and it has been successfully demonstrated that delivery systems can pass through the intestinal mucosa whilst protecting the encapsulated small molecules, peptides, proteins, nucleic acids, or vaccines, to ensure their absorption [10–14]. A large number of studies have shown that appropriate nano system selection and fine-tuning of the physical and chemical properties of the system can enhance drug absorption. In addition to preventing degradation by enzymes and acids in the gastrointestinal tract and increasing the solubility of drugs in the lumen, delivery carriers can also enhance transport through the gastrointestinal barrier [15–17].

Polymer nanoparticles have many advantages over other delivery platforms. Compared with single-unit preparations, multiarticulate systems such as nanoparticles are more evenly distributed in the gastrointestinal tract, which results in more uniform drug absorption and reduced risk of local irritation [18, 19]. Nanoparticle size is thought to be a key parameter as microparticles larger than 10 μm are unable to penetrate the mucus layer. In addition, the absorption of nanoparticles by intestinal cells is better than that of microparticles; and for particles larger than 4 μm, absorption becomes notably challenging. Nanoparticles are also more stable in biological fluids than liposomes—which are widely applied as drug carriers [11, 20]. Liposomal intestinal cells are difficult to maintain intact during endocytosis, which limits the potential applications of these carriers.

From a pharmacological perspective, the choice of polymeric material can be used to control physical and chemical properties (for example: hydrophobicity, zeta potential), drug release characteristics (for example: delay, prolongation, triggers), and biological behavior (for example: targeting, bio adhesion, improved cell absorption) [21–24]. From a physiological point of view, the gastrointestinal barrier is designed to protect our bodies from foreign compounds. The therapeutic agent must first diffuse through the mucous layer, and then enter the blood or lymph circulation through the epithelial cell barrier under the mucosa [15, 25]. However, thorough research on the actual outcomes for NPs in the digestive tract remains limited [11].

This study aims to evaluate the impact of particle size, surface charge, and surface hydrophilicity/hydrophobicity—the basic physical properties of polymer nanoparticles—on their ability to overcome the intestinal barrier and achieve oral absorption, by constructing corresponding model nanoparticles with a single variable. The performance of these model NPs in each stage of the intestinal absorption process is specifically evaluated, including the ability of NPs to penetrate the small intestinal mucus layer and the ability to penetrate the small intestinal cell barrier.

Owing to the dispersion stability and uniform controllable size of fluorescently labeled polystyrene nanoparticles (PSNPs) in various dispersion media, they were selected as the model particles for evaluating the effect of particle size [21, 26, 27]. Grafted water-soluble chitosan NPs that can be prepared with controlled particle size and surface charge served as model particles for evaluating surface charge; including negatively charged grafted carboxymethyl chitosan NPs (CMCNPs) and positively charged grafted chitosan hydrochloride NPs (CHNPs), rhodamine B (RhB) was used as covalent label for these NPs [28–30]. Poly(lactic-co-glycolic acid) (PLGA) with different monomer ratios can be used to prepare NPs with different degrees of surface hydrophobicity. NPs made using PLGA-polyethylene glycol (PEG)-PLGA triblock copolymer present a dense hydrophilic layer at the surface [31–34]. These (PEG-)PLGA nanoparticles served as hydrophilic models. Coumarin 6 (C6) and Nile red (NR) were used to label the NPs.

The model NPs were characterized and then evaluated using a single factor-controlled investigation. Various experimental models—based on the small intestinal mucus barrier that prevents the passage of macromolecules and the selective monolayer small intestinal cell barrier [35] —were used to evaluate and summarize the ability of NPs with different basic physical properties to overcome the physiological barrier to intestinal absorption.

## Materials and methods

### Material and equipment

Fluorescent polystyrene NPs (Flu-PSNPs) were purchased from Tianjin BaseLine chromatography technology development center (Tianjin, China). Carboxymethyl chitosan and chitosan hydrochloride (molecular weight, 100 kDa) were purchased from Jinke Biochemical Co. Ltd. (Zhejiang, China). All PLGA and PLGA-PEG-PLGA triblock copolymers were purchased from Jinan Daigang Biomaterial Co., Ltd.; C6, RhB, methyl-β-cyclodextrin (M-β-CD), chlorpromazine hydrochloride, 5-(*N*-ethyl-*N*-isopropyl) amiloride (EIPA), and formalin were purchased from Shanghai Aladdin Reagent Co. Ltd. (Shanghai, China). 4,6-diamino-2-phenyl indole (DAPI), brefeldin A, monensin, and bafilomycin A1 were obtained from Dalian Meilun Biotechnology Co., Ltd. (Dalian, Liaoning, China). Lysosome (Lyso)-Tracker probe, endoplasmic reticulum (ER)-Tracker probe, and golgi complex (Golgi)-Tracker were purchased from KeyGEN Biotech (Nanjing, Jiangsu, Biotechnology (Shanghai, China). *N*-acetyl-l-cysteine (NAC) was purchased from Shanghai Macklin Biochemical Co., Ltd. (Shanghai, China). Caco-2 cells were from Cell Resource Center, Shanghai Institute of Life Sciences, Chinese Academy of Sciences (Shanghai, China). Sprague–Dawley (SD) rats were obtained from Jinan Pengyue Experimental Animal Breeding Co., Ltd. (Jinan, China).

### Preparation of the model nanoparticles

NPs with different surface charge were prepared via grafting polymerization [30, 36]. Carboxymethyl chitosan or chitosan hydrochloride was dissolved in 100 mL of deionized water in a 40 °C water bath under nitrogen over 30 min. After adding methyl methacrylate (MMA), ammonium persulfate (APS) solution was added dropwise at 75 °C and allowed to react for 5 h, then the particles were separated by centrifugation. The grafting of hydrophobic MMA onto the hydrophilic chitosan deionizer resulted in the formation of NPs from the amphiphilic polymer.

RhB(4 mg) was added to 10 mL of pH 4.5 hydrochloric acid solution and allowed to dissolve. 1-Ethyl-3-(3-dimethylaminopropyl)carbodiimide hydrochloride (EDC·HCl, 2 mg) and *N*-hydroxysuccinimide (NHS, 2 mg) were then added and the reaction mixture was stirred for 2 h in the dark. CMCNPs or CHNPs suspension (30 mL) was then added and the mixture was stirred for 24 h in the dark. The reaction product was dialyzed against pure water until no fluorescence was detected in the dialysis medium to obtain RhB-labeled grafted water-soluble chitosan nanoparticles (RhB-CMCNPs and RhB-CHNPs). Adjusting the input ratio (Table 1) of raw materials and initiator in the preparation process allowed three kinds of fluorescent CMCNPs and CHNPs with different final surface charges to be obtained.

**Table 1:** Partial characterization of CMCNPs and CHNPs (n = 3)

Nanoparticles group	CMC, CH:MMA Molar ratio (w/w)	Initiator dosage(mg)	Particle size (nm)	Polydispersity Index	Zeta potential (mV)
CMCNPs (-40)	3:1	150	163.6 ± 7.9	0.081 ± 0.006	− 18.80 ± 3.2
CMCNPs (-30)	1:1	30	166.7 ± 7.5	0.066 ± 0.011	− 27.97 ± 4.0
CMCNPs (-20)	1:5	30	169.2 ± 8.9	0.108 ± 0.006	− 43.40 ± 3.9
CHNPs (+20)	1:1	300	144.3 ± 4.4	0.075 ± 0.007	16.09 ± 5.1
CHNPs (+30)	1:1	70	158.3 ± 5.0	0.006 ± 0.002	31.39 ± 4.2
CHNPs (+40)	1:4	50	144.9 ± 5.7	0.070 ± 0.015	40.27 ± 3.8

(PEG-)PLGA nanoparticles were prepared using the emulsification solvent evaporation method [37, 38]. One hundred milligrams of PLGA (20000^a^, 50:50^b^), PLGA (20000^a^, 75:25^b^), PLGA (20000^a^, 85:15^b^) or triblock copolymer PLGA (20000^a^, 50:50^b^)-PEG (550^c^)-PLGA (20000^a^, 50:50^b^), PLGA(20000^a^, 50:50^b^)-PEG(1000^c^)-PLGA(20000^a^, 50:50^b^), PLGA(20000^a^,50:50^b^)-PEG(2000^c^)-PLGA(20000^a^,50:50^b^), and 0.2 mg of C6 or NR were dissolved in 2 mL of dichloromethane-ethyl acetate (7:3) mixed solvent to obtain the organic phase. The organic phase was added to 8 mL of 3% (w/v) polyvinyl alcohol (PVA) aqueous solution, and sonicated for 100 s under 200 W sonication (2-s pulses, 1-s breaks) in an ice bath to form an emulsion (o/w). The emulsion was then added dropwise to 50 mL of 0.5% PVA solution and magnetically stirred for 3 h to evaporate the organic solvent to form nanoparticles with encapsulated C6 or NR (C6-PNPs, C6-PPNPs, NR-PNPs, and NR-PPNPs). The NPs were then dialyzed for 48 h before collecting by centrifugation. (^a^ represents the average molecular weight of PLGA, ^b^ represents the ratio of LA to GA in the PLGA chain, and ^c^ represents the average molecular weight of PEG)

### Characterization of model nanoparticles

A dynamic light scattering (DLS) laser particle size analyzer (Delsa™ Nano C, Beckman Coulter, USA) was used to measure the average hydrodynamic diameter, polydispersity index (PDI), and zeta potential values of all CMCNPs, CHNPs, PNPs, and PPNPs at room temperature. All samples were measured three times. A Phenom XL scanning electron microscope (SEM) system (Phenom, The Netherlands) was used to observe the surface morphology of the PSNPs. A JEM-1230 transmission electron microscope (TEM) system (Jeol, Tokyo, Japan) was used to observe all PSNPs, as well as the to determine the morphology of the CMCNPs, CHNPs, PNPs, and PPNPs. The contact angle of all PNPs and PPNPs were measured with a contact angle measuring instrument (JC 2000D 2A, Shanghai Zhongchen, China) after freeze-dried and pressed into tablets. Besides, the PNPs or PPNPs were dispersed in 1 mL double-distilled water saturated with *n*-octanol and added to a vial. Four milliliters of water-saturated *n*-octanol was then added and the sample was vortexed for 3 min and then left to oscillate at 30 °C for 1 h. The fluorescence intensities of the aqueous phase and the organic phase were then measured, and the Pow values of the NPs were calculated using the following formula.

$$ {\text{Pow }} = {\text{ F}}_{\text{o}} /{\text{F}}_{\text{w}} $$.

F_o_ is the equilibrium fluorescence intensity of NPs in *n-*octanol, and F_w_ is the equilibrium fluorescence intensity of NPs in the water phase.

Finally, all Flu-PSNPs, RhB-CMCNPs, RhB-CHNPs, C6-PNPs, C6-PPNPs NR-PNPs and NR-PPNPs were dispersed and incubated in deionized water, phosphate buffered saline (PBS), and artificial intestinal fluid. The fluorescence intensities were measured at 0, 4, and 24 h. The fluorescence intensity changes were calculated using to the following formula.

$$ {\text{Fluorescence intensity change }} = \, \left( {{\text{F}}_{\text{t}} - {\text{ F}}_{0} } \right)/{\text{F}}_{0} $$

F_0_ is the fluorescence intensity of fluorescent NPs when t = 0 h and F_t_ is the fluorescence intensity of fluorescent NPs when t = 4 or 24 h after dispersed in different media.

### In vitro intestine penetration

SD rats were fasted overnight and anesthetized with 20% urethane (5 mL/kg). The abdomen was cut at the midline and the small intestine was taken out and divided into 9-cm segments. After the mesentery and lymph nodes were removed, the intestinal ring was turned over, and the inner surface of the intestine was cleaned. Tyrode’s solution (2 mL, 37 °C) was injected into the sac and both ends of the intestinal segment were tied tightly. The everted intestinal sacs were put into Tyrode’s solution containing each group of fluorescent NPs and incubated in at 37 °C in a 5% CO_2_ incubator for 3 h. Air was injected with a syringe every 15 min to maintain the oxygen content. The fluorescence signal value of the liquid in the capsule was measured after incubation.

### In situ intestine absorption

After fasting, SD rats were anesthetized and then a laparotomy was conducted to expose the small intestine. A 4-cm intestinal ring was made by ligating each end. Each group of fluorescent NPs was injected into an intestinal ring and incubated for 2 h. Subsequently, all intestinal rings were thoroughly washed to remove residual NPs. After weighing, the intestinal ring was homogenized using a high-speed shearing machine, and the fluorescence signal of the sample was quantitatively measured [39].

In addition, after the intestinal ring was incubated with NPs for 3 h, the rats were euthanized, and the ligated intestinal segment was removed, washed, and immersed in 4% formalin solution for fixation. The intestinal ring was then dehydrated, sectioned in a cryostat, and stained with DAPI. Anti-fluorescence quencher was used to prevent fluorescence quenching of the section. A live cell imager was used for slice imaging.

### Transmucus transport

Fresh mucus from the small intestine of a rat was pipetted into the lumen of the Transwell, spread evenly, and equilibrated at 37 °C for 30 min in a 95% relative humidity environment. Hank’s balanced salt solution (HBSS, 600 μL) was added to the receptor compartment and allowed to equilibrate for 15 min. Next, 500 μL of CMCNPs, CHNPs, PNPs or PPNPs was added carefully to the donor side. The device was then incubated in the above conditions. At 15, 30, 60, 90, and 120 min, 200-μL aliquots were taken from the receptor side and replaced with 200 μL of fresh HBSS. The apparent permeability coefficient (P_app_) was calculated using the formula:

$$ {\text{P}}_{\text{app}} = {\text{ dQ}}/{\text{dt}} \times 1/({\text{A}} \times {\text{C}}_{0} ), $$

where dQ/dt is the NPs flux from the donor to the acceptor; C_0_ is the initial concentration of NPs on the donor side, and A is the membrane area (cm ^2^).

Simultaneously, after incubating with mucus for 2 h, the mucus layer was carefully removed and the relative amount of fluorescence accumulation in the mucus was measured [13].

In addition, NAC is a mucolytic agent widely used in the clinic that can destroy the hydrogen bonds and disulfide bonds of mucus [14]. Based on the procedure described in section “In situ intestine absorption”, the intestinal ring was treated with 0.2% NAC to remove the mucus layer before and after incubation with the fluorescent nanoparticles. The pre-incubation treatment served as the pretreatment group, the post-incubation treatment served as the post-treatment group, and the untreated intestinal ring served as the control group. The other processing methods were as described in section “In situ intestine absorption”, and the sample was measured.

### Cellular uptake

Caco-2 and HT29-MTX cells were used for cell culture [5]. The cells were grown in a 21-cm^2^ cell culture dish containing Dulbecco’s modified eagle media (DMEM) supplemented with 10% fetal bovine serum, 1% non-essential amino acids, 1% penicillin and streptomycin. The cells were cultured at 37 °C with 90% relative humidity and 5% CO_2_ supply.

A co-culture of Caco-2 and HT29-MTX cells (8:2) was used as an in vitro model to simulate the intestinal mucus and epithelial environment [6]. The cells were seeded in a 24-well plate at a density of 3 × 10^4^ cells/well and placed in an incubator for 14 days. The original medium was then replaced with each group of fluorescent NPs (10 μg/mL), and cells were incubated for a further 3 h. After the treatment the cells were washed three times with PBS, and the cells were stained with DAPI to indicate the nuclei. Cells were subsequently fixed by adding 500 μL of 4% paraformaldehyde for 15 min. The cells were washed three times with PBS and observed using a high-resolution live cell imaging system (BioTek Cytation™ 5, US).

The Caco-2 cells were seeded in a 6-well cell culture plate at a rate of 3 × 10^5^ cells/well and incubated for 14 days. Cells were treated with fluorescent NPs (20 μg/mL) for 1.5 h. The cells were then trypsinized and centrifuged at 2000×*g* for 5 min. The supernatant was discarded and the cells were washed three times with PBS. Subsequently, the cells were resuspended in 600 μL of fresh PBS and analyzed by flow cytometry (BD Accuri C6, US).

In addition, the cell internalization mechanism of model NPs was studied by adding different specific cellular pathway inhibitors. Caco-2 cells in the logarithmic growth phase were seeded at 3 × 10^5^ cells/well in a 6-well plate and grown for 48 h. The following inhibitors (2 mL) were then added: chlorpromazine (30 μmol/L), EIPA (20 μmol/L), formalin (20 μmol/L), and M-β-CD (2.5 mmol/L). After 0.5 h, fluorescent NPs (20 μg/mL) were added to the co-cultured cells, which were then incubated for 1 h. Following treatment as described above, the fluorescence intensity of the cells was measured by flow cytometry [7, 8]. For the control group PBS was used instead of an inhibitor.

Everted rat intestinal sacs were prepared using the method described in section “In vitro intestine penetration”. The everted intestinal segments were then incubated for 30 min in Tyrode’s buffer containing the following inhibitors: formalin (20 μmol/L), chlorpromazine (30 μmol/L), EIPA (20 μmol/L), and M-β-CD (2.5 mmol/L). Subsequently, the extracapsular solution was replaced with suspensions containing each group of fluorescent NPs, in addition to the corresponding inhibitors at the same concentration. The control group did not contain inhibitors. Samples were incubated in a 37 °C, 5% CO_2_ incubator for 3 h, and air was injected with a syringe every 15 min to maintain the oxygen content [40]. The fluorescence intensity of the liquid in the capsule was measured after incubation.

### Intracellular transport

The fate of the model NPs after entering the cells was tracked by organelle colocalization [9, 10]. Caco-2 cells were seeded in a 24-well plate at a density of 3 × 10^4^ cells/well and placed in an incubator for 48 h. NPs were then added carefully and incubated with cells at 37 °C for 2 h. After incubation, the cells were rinsed with cold PBS, washed thoroughly and fixed with 4% paraformaldehyde. The cells were treated with Lyso-Tracker, ER-Tracker, and Golgi-Tracker and then DAPI was added for nuclear staining. The samples were observed using a high-resolution live cell imaging system.

In addition, Caco-2 cells were seeded in a 6-well plate at 3 × 10^5^ cells/well and grown for 48 h. The cells were then incubated with NPs (20 μg/mL) for 1 h. The suspension was replaced with culture medium containing bafilomycin A1 (100 nM), monensin (32.5 µg/mL), and brefeldin A (25 µg/mL) and incubation was continued for 2 h. Following treatment as described in the “Cellular uptake” section, the cell suspension was analyzed by flow cytometry [11]. Cells incubated with NPs only served as the blank group, and cells incubated with medium without inhibitor served as the control group.

### Transcellular transport

Caco-2 cells and HT29-MTX cells (8:1) were seeded on a perforated plate (Corning Transwell 3460) at a concentration of 2 × 10^5^ cells/well, and the medium in the upper and lower compartments was changed every day. A cell resistance meter (ERS; Millipore Corp., Bedford, MA, USA) was used to measure the transepithelial resistance (TEER) value to monitor the integrity of the cell monolayer. After 21 days of cell differentiation, a monolayer with a TEER value higher than 300 Ω/cm^2^ was used. The cell monolayer was equilibrated in HBSS at 37 °C. After equilibration, the cell monolayer was incubated with each of the CMCNPs, CHNPs, PNPs, and PPNPs groups at 37 °C. At 15, 30, 60, 90, and 120 min, 200-μL samples were taken from the basolateral chamber and replaced with an equal volume of HBSS. The transport volume was measured with a microplate reader and the cumulative transport volume was calculated. Each experiment was repeated three times [41]. The samples were then measured and the formula given in the “Transmucus transport” section was used to calculate the P_app_ value.

### Statistical analysis

The statistical significance of the results was analyzed using a two-tailed student’s t-test. A p-value < 0.05 (p < 0.05) was considered statistically significant.

## Results

### Characterization of model NPs

**Fig. 1:**
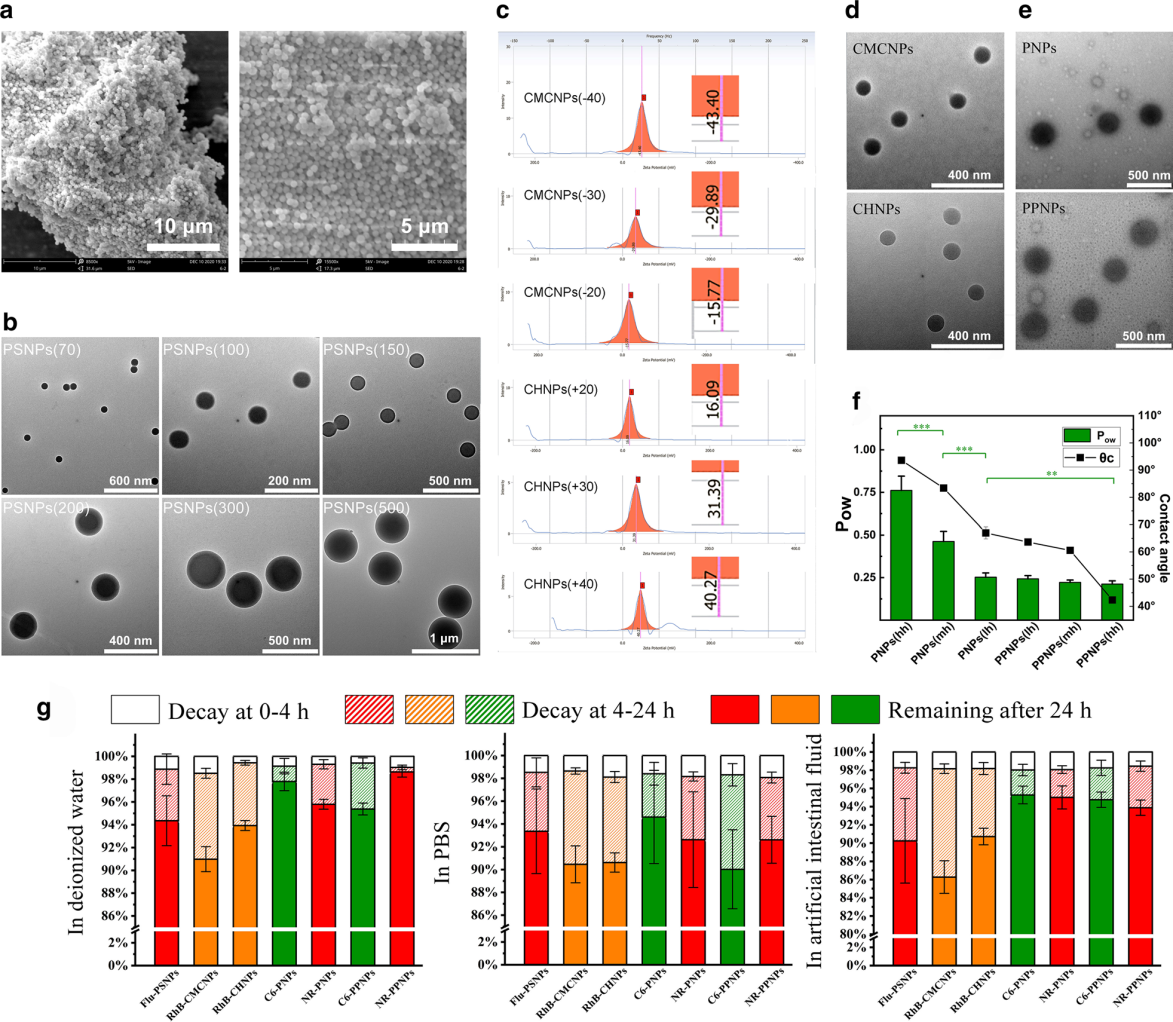
Characterization of model NPs: **a** SEM micrographs of PSNPs. **b** TEM micrographs of PSNPs in each group. **c** ζ potential of CMCNPs and CHNPs in each group. **d** TEM micrographs of CMCNPs and CHNPs. (e) TEM micrographs of PNPs and PPNPs. **f** Evaluation of hydrophilicity of PNPs and PPNPs in each group (data are mean ± SD, n = 5, **p < 0.01, ***p < 0.001). **g** Fluorescence stability of model NPs in each group

In this study, polystyrene nanoparticles, water-soluble chitosan nanoparticles, and (PEG-)PLGA nanoparticles were used as model NPs representing the particle size group, zeta potential group, and surface hydrophobicity/hydrophilicity group, respectively. The PSNPs had regular spherical morphology (Fig. 1a) and uniform particle sizes of approximately 70, 100, 150, 200, 300, and 500 nm (Fig. 1b). The three CMCNPs and three CHNPs used different raw material and initiator (APS) input ratios; however, the average particle sizes determined by DLS measurement were in the range 155 ± 15 nm (Table 1), with approximate zeta potential values of −20, −30, −40, +20, +30, and +40 mV (Fig. 1c). The CMCNPs and CHNPs showed similar morphology in the TEM images (Fig. 1d). The (PEG-)PLGA nanoparticles made of different materials showed similar particle size, zeta potential (Table 2), and spherical morphology (Fig. 1e); however, contact angle and lipid–water distribution studies showed that they had different surface hydrophilicity/hydrophobicity (Fig. 1f). All of the fluorescently labeled nanoparticles were tested for their fluorescence stability in deionized water, PBS, and artificial intestinal fluid (Fig. 1g). The fluorescence changes exhibited after 4 h were all within 3%, and most NPs changed within 10% after 24 h. There was also no significant difference between the fluorescence of all fluorescent NPs in various media at 4 h and 0 h.

**Table 2:** Partial characterization of PNPs and PPNPs (n = 3)

Nanoparticles group	Ratio of LA and GA	Hydrophilic material attached	Particle size (nm)	Polydispersity Index	Zeta potential (mV)	Contact angle (°)
PNPs (hh)	85:15	None	195.6 ± 4.2	0.103 ± 0.013	− 0.33 ± 0. 25	93.60 ± 1.24
PNPs (mh)	75:25	None	189.6 ± 6.8	0.117 ± 0.026	− 0.69 ± 0.02	83.38 ± 0.07
PNPs (lh)	50:50	None	203.6 ± 2.3	0.066 ± 0.007	− 1.63 ± 0.07	66.98 ± 2.21
PPNPs (lh)	50:50	PEG550	202.5 ± 7.4	0.070 ± 0.025	− 1.29 ± 0.82	63.65 ± 0.04
PPNPs (mh)	50:50	PEG1000	189.7 ± 1.9	0.082 ± 0.016	− 4.23 ± 2.23	60.57 ± 0.03 42.32 ± 0.50
PPNPs (hh)	50:50	PEG2000	191.3 ± 7.5	0.098 ± 0.039	− 1.32 ± 1.05	

According to their different particle sizes, the fluorescent polystyrene particles were denoted PSNPs (70), PSNPs (100), PSNPs (150), PSNPs (200), PSNPs (300), and PSNPs (500). Of the six grafted water-soluble chitosan nanoparticles obtained, three had different positive charges and three had different negative charges, they were denoted CMCNPs (−20), CMCNPs (−30), CMCNPs (−40), CHNPs (+20), CHNPs (+30) and CHNPs (+40). The NPs prepared from the six different (PEG-)PLGA materials were named as PNP (hh), PNP (mh), PNP (lh), PPNP (lh), PPNP (mh), and PPNP (hh) corresponding to high hydrophobicity, medium hydrophobicity, low hydrophobicity, low hydrophilicity, medium hydrophilicity, and high hydrophilicity respectively according to their hydrophobicity/hydrophilicity.

### Intestinal absorption of model NPs

**Fig. 2:**
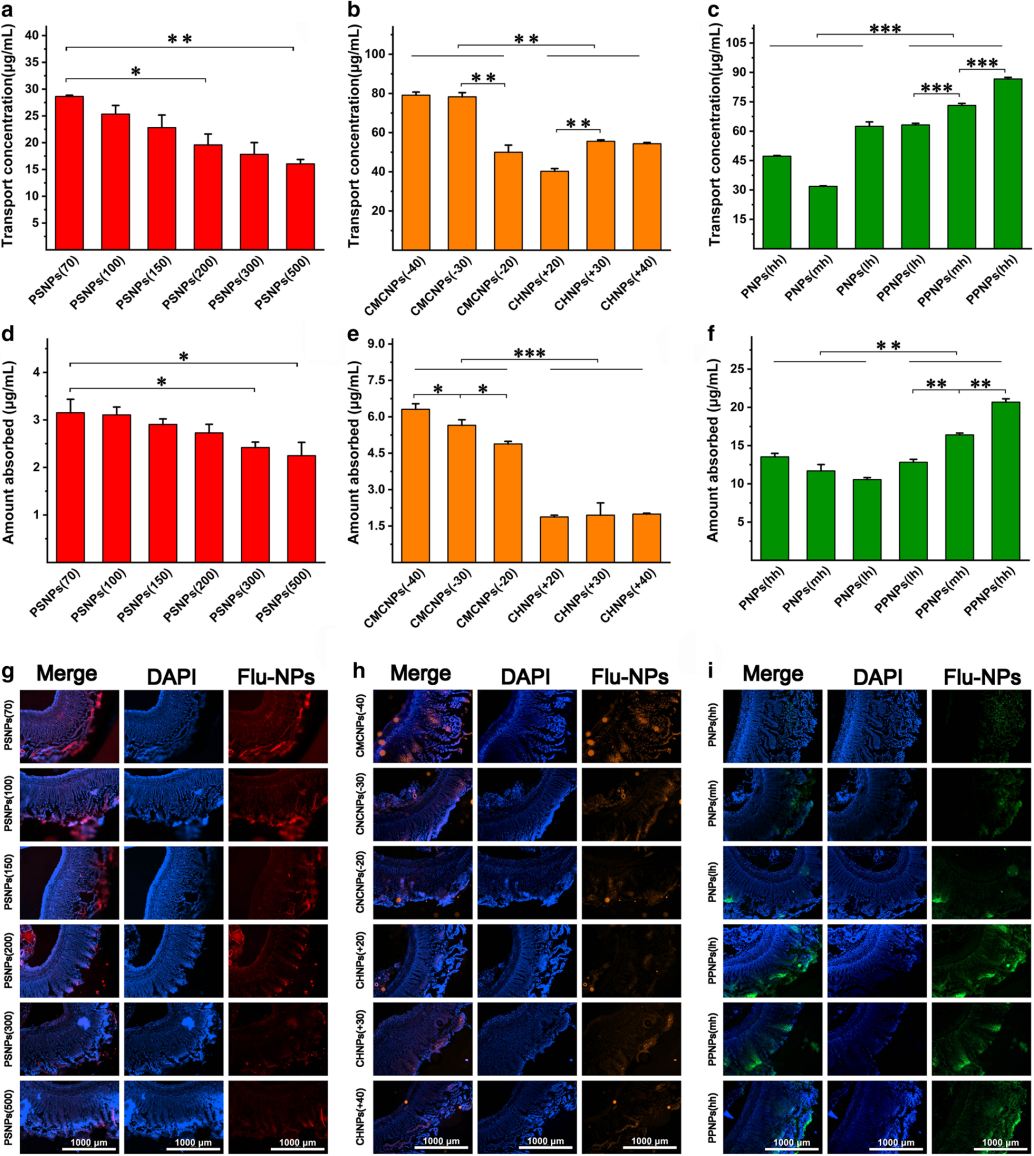
Intestinal absorption evaluation of model NPs: Evaluation of the intestinal permeation ability of PSNPs (**a**), CMCNPs, CHNPs (**b**), PNPs and PPNPs (**c**) in the eversion intestinal sac model (data are mean ± SD, n = 3, *p < 0.05, **p < 0.01, ***p < 0.001). Evaluation of the intestinal permeation ability of PSNPs (**d**), CMCNPs, CHNPs (**e**), PNPs and PPNPs (**f**) in the rat in situ intestine ring model (data are mean ± SD, n = 3, *p < 0.05, **p < 0.01, ***p < 0.001). Fluorescence imaging of PSNPs (**g**), CMCNPs, CHNPs (**h**), PNPs and PPNPs (**i**) uptaken in intestinal sections

The everted intestinal sac model constructed from the isolated small intestine of rats was used to evaluated the NPs. The permeability of the small intestine is shown in Fig. 2a–c. It can be seen that the permeability of the isolated small intestine tissue to NPs was size-dependent. The amount of NPs with a particle size of 100 nm that penetrated the intestine was 1.44 times that of NPs with a particle size of 500 nm. In addition, for the CMCNPs and CHNPs used in this study, the amount of CMCNPs—which had a negative surface charge—that penetrated the intestine was 1.38 times that for CHNPs—which had a positive surface charge. Comparing NPs with similar charge magnitude showed that the penetrations of all of the negatively charged NP groups were greater than those of the positively charged NP groups. For the PLGA nanoparticles, the PPNPs—which had hydrophilic surfaces—showed 1.58 times the penetration of PNPs without PEG. In addition, the longer the PEG chain used (within the scope of this study), the greater the penetration of the PPNPs.

The small intestine absorption of the model nanoparticles was quantitatively evaluated using a rat in vivo intestinal ring model (Fig. 2d–f), and the intestinal rings incubated with the NPs samples were imaged by sectioning (Fig. 2g–i). Similar to the findings for penetration in the isolated small intestine, the absorption of NPs in the in vivo model showed size dependence. The absorption of NPs with a particle size of 100 nm was 1.38 times that of NPs with a particle size of 500 nm. In addition, in the in vivo intestinal ring absorption model, the negatively charged CMCNPs showed 2.9 times the absorption of the positively charged CHNPs, and model NPs with a higher degree of negative surface charge exhibited greater absorption or adsorption values. For the PNPs and PPNPs, the hydrophilic PPNPs showed in vivo absorption or adsorption values that were 1.4 times those of the PNPs, and longer PEG chains resulted in increased absorption.

Drugs and preparations must overcome the corresponding biological barrier after administration to complete the absorption process. Generally, the absorption site for orally administered drugs is the small intestine, and the main two absorption barriers of the small intestine are the small intestinal mucus layer and small intestinal epithelial cells connected by tight junctions. First, drug carriers—including polymer nanoparticles—may adhere to the mucus, which prevents them from easily penetrating the mucus layer. In addition, if the nanoparticles are absorbed in the small intestine epithelium monolayer through the transcellular pathway, they need to undergo a unidirectional process of endocytosis and exocytosis, that is, the intestinal lumen side enters the cell, the intracellular polarity is transferred, and basolateral exocytosis [42]. The intestinal absorption of nanoparticles must overcome these obstacles in turn. Therefore, this study also conducted an in-depth evaluation of the differences in the various stages of intestinal absorption for model NPs with different properties.

### Evaluation of model NPs permeation in the mucus layer

The small intestinal mucosa is a physical and chemical barrier that can hinder drug absorption. Although it is generally believed that the epithelial structure that is densely packed with active cells is the key barrier and the limiting factor in determining the systemic absorption of drug molecules, other non-epithelial mucosal barrier components still need to be characterized [43]. This is particularly true for nano formulations that require mucosal administration. It is necessary to fully understand the mucosal barrier to promote the development of drug delivery technology [44, 45].

**Fig. 3:**
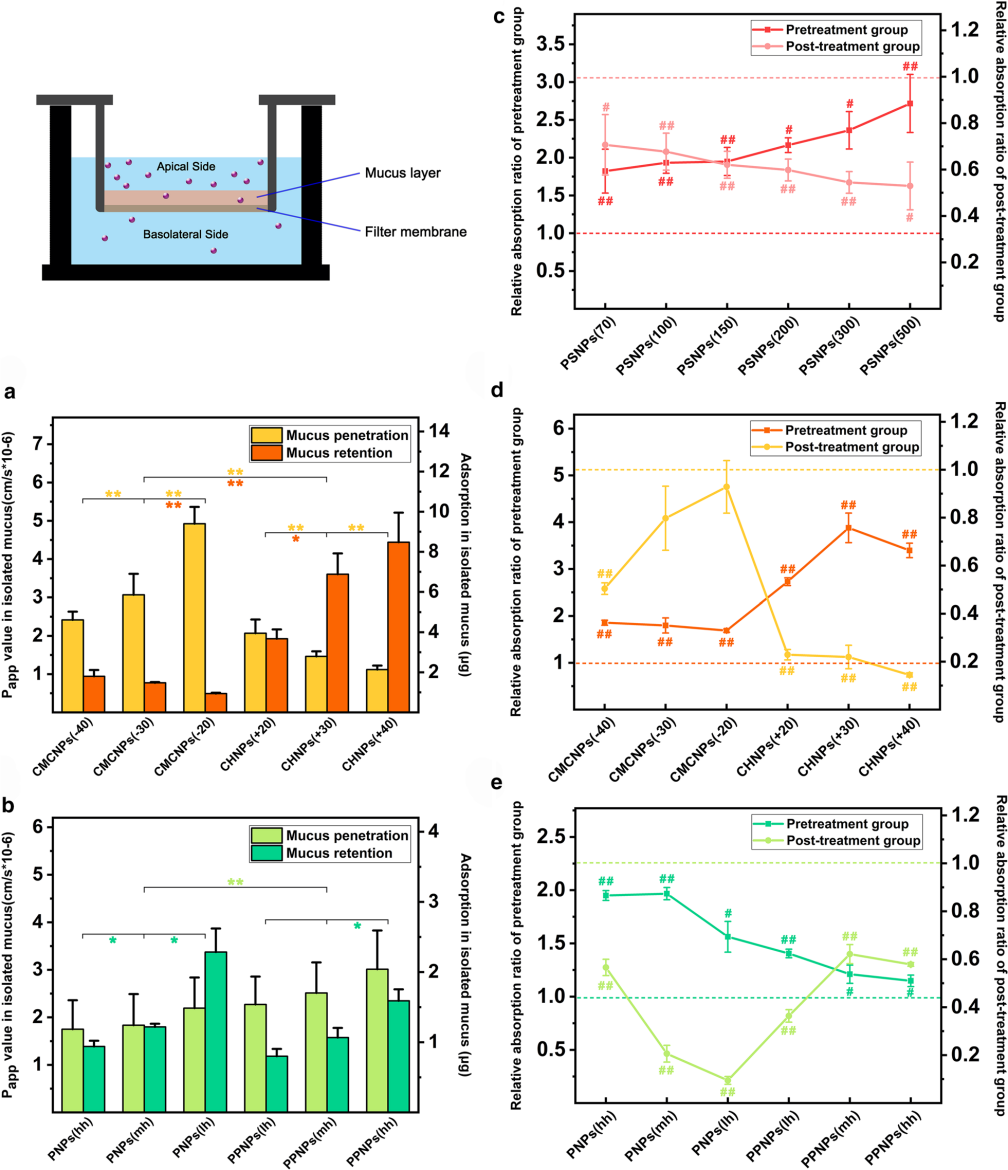
Evaluation of the ability of model NPs to penetrate the mucus layer: Evaluation of CMCNPs, CHNPs (**a**), PNPs and PPNPs (**b**) permeation and retention in isolated intestinal mucus (data are mean ± SD, n = 3, *p < 0.05, **p < 0.01). Research on the interaction between PSNPs (**c**), CMCNPs, CHNPs (**d**), PNPs and PPNPs (**e**) and mucus layer by removing mucus in the rat in situ intestine ring model (data are mean ± SD, n = 3, Compared with a control group that was not treated with mucus removal, ^#^p < 0.05, ^##^p < 0.01)

To explore the penetration of NPs with different properties in the small intestinal mucus, isolated rat small intestinal mucus was spread in a Transwell. The apparent permeability coefficient values of all of the varied charge and hydrophobicity model NPs were determined at several preset time points as shown in Fig. 3a–b. Compared with the positively charged CHNPs, the negatively charged CMCNPs were significantly easier to transport from one side of the mucus to the other. In addition, regardless of whether the surface charge was positive or negative, NPs with a lower charge magnitude showed higher transport levels. The apparent permeability coefficient of CMCNPs (−20) was approximately twice that of CMCNPs (−40), and CHNPs (+20) had a P_app_ value of approximately 1.85 times that of CHNPs (+40). The average P_app_ values of the PNPs and PPNPs showed a positive correlation with hydrophilicity, and PPNP (hh)—which had the longest PEG chains—exhibited the best transport results.

The isolated rat small intestinal mucus was removed from the Transwell after the 2-h incubation with each CMCNP, CHNP, PNP, and PPNPs group, and the fluorescence intensity was measured to investigate the retention of NPs in the mucus. The experimental results (Fig. 3a, b) show that the overall mucus retention of the positively charged CHNPs was 4.51 times of that of the negatively charged CMCNPs. In addition, the model NPs used in this experiment showed the tendency that smaller zeta potential magnitude led to lower mucus retention, regardless of whether the charge was positive or negative. For the NPs used to study hydrophilicity/hydrophobicity, the retention of PNPs was 1.29 times that of the PPNPs. The PPNPs—which contained PEG—showed a lower retention rate as a whole, and there was a tendency for retention rate to increase with increasing PEG chain length. The mucus retention of PNPs without a PEG component was positively correlated with the hydrophilicity of the material, and the mucus retention of PNP (lh) was 2.6 times that of PNP (hh).

To further understand the interaction between NPs and small intestinal mucus for the different models—using the intestinal ring model in rats as a basis—NAC was used before incubation with NPs (pretreatment group) and after incubation (post-treatment group) to relax and release the viscosity of the small intestine mucus. The absorption results were then measured and plotted (Fig. 3c–e). In the pretreatment group, the absorption of all NPs increased, and the degree of improvement showed a positive correlation with particle size. The absorption of PSNPs (70), PSNPs (100), PSNPs (150), PSNPs (200), PSNPs (300), and PSNPs (500) increased to 1.82, 1.93, 1.95, 2.17, 2.36, and 2.36 times the original, respectively. The model NPs with positive surface charge and hydrophobic surfaces also showed improved absorption. The absorption of CMCNPs (−40), CMCNPs (−30), CMCNPs (−20), CHNPs (+20), CHNPs (+30), and CHNPs (+40) increased to 1.86, 1.80, 1.69, 2.73, 3.88, and 3.40 times the original, respectively. In addition, the absorption of PNP (hh), PNP (mh), PNP (lh), PPNP (lh), PPNP (mh), and PPNP (hh) increased to 1.95, 1.97, 1.56, 1.41, 1.21, and 1.15 times the original, respectively. When the small intestinal mucus was removed and analyzed after the intestines had been incubated with NPs, the small NPs were found to exhibit the greatest retention. The absorption of PSNPs (70), PSNPs (100), PSNPs (150), PSNPs (200), PSNPs (300), and PSNPs (500) reduced by 29%, 32%, 38%, 40%, 46%, and 47%, respectively. Negative surface charge and greater charge magnitude made the NPs more likely to be retained. The absorption of CMCNPs (−40), CMCNPs (−30), CMCNPs (−20), CHNPs (+20), CHNPs (+30), and CHNPs (+40) reduced by 50%, 20%, 7%, 77%, 78%, and 85%, respectively. For the PNPs and PPNPs groups, PNP (lh), which did not contain PEG but was more hydrophilic than the other two PNPs, had the lowest remaining penetration. The absorption of PNP (hh), PNP (mh), PNP (lh), PPNP (lh), PPNP (mh), and PPNP (hh) reduced by 43%, 79%, 91%, 64%, 38%, and 42%, respectively.

### Evaluation of model NPs permeation in monolayer cells

The intestinal epithelial cell layer is the last barrier to intestinal absorption. These cells are cylindrical epithelial cells with a brush-like border membrane on the top, covered by glycocalyx—a dense grid of sugar structures—and connected to each other by tight junctions. Theoretically, macromolecules or particles can traverse the intestinal epithelium through transcellular pathways as well as tight junction pathways. Of these two, transcellular pathways are the most studied and is the main focus of this study. The transcellular transport of nanoparticles starts with endocytosis in the apical membrane of the cell, which is the process by which the particles are absorbed into the cell [46]. The particles are then transported through the cell and released at the basolateral membrane.

**Fig. 4:**
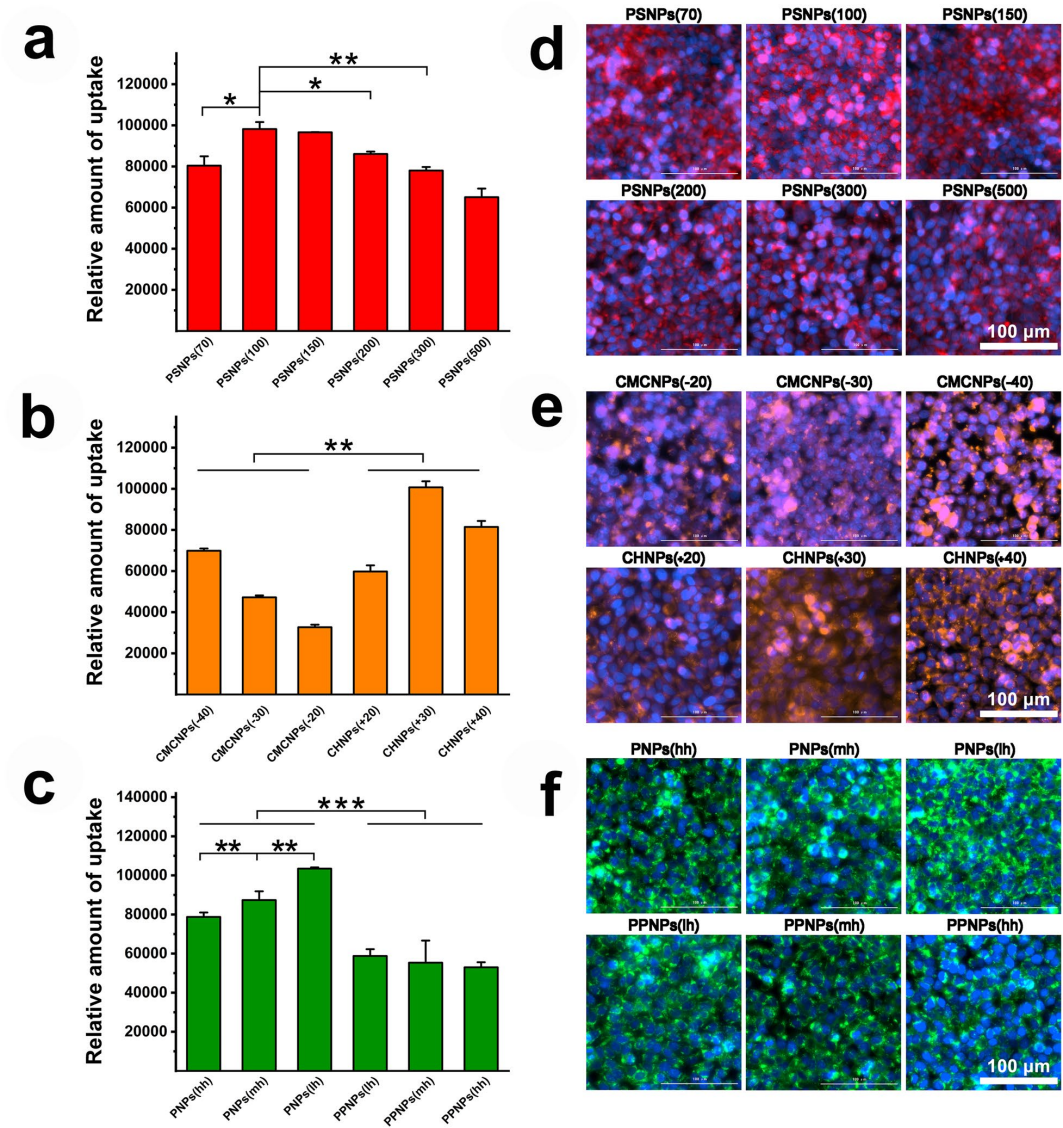
Evaluation of cellular uptake of model nanoparticles: Uptake of PSNPs (**a**), CMCNPs, CHNPs (**b**), PNPs and PPNPs (**c**) by mixed-cultured caco-2 and HT29-MTX cells (data are mean ± SD, n = 3, *p < 0.05, **p < 0.01, ***p < 0.001). Imaging of fluorescent PSNPs (**d**), CMCNPs, CHNPs (**e**), PNPs and PPNPs (**f**) absorbed by mixed-cultured caco-2 and HT29-MTX cells

Each model NPs group was incubated with a co-culture system of Caco-2 and HT29-MTX cells that had formed a monolayer and fully differentiated. Quantification of cell uptake (Fig. 4a–c) and imaging analysis (Fig. 4d–f) were conducted. Of the PSNPs groups, PSNPs (100) showed the highest uptake, which was 1.22 times that of PSNPs (70) and 1.51 times that of PSNPs (500). The overall uptake of CHNPs was 1.62 times of CMCNPs. Comparing particles with the same charge magnitude, NPs with positive charge had higher absorption than NPs with negative charge. Comparing PNPs and PPNPs, the PEG component was found to reduce the cellular uptake of NPs. The overall uptake of PPNPs was approximately 62% that of PNPs. Additionally, PNPs (lh) with no PEG component modification had the highest cellular uptake.

**Fig. 5:**
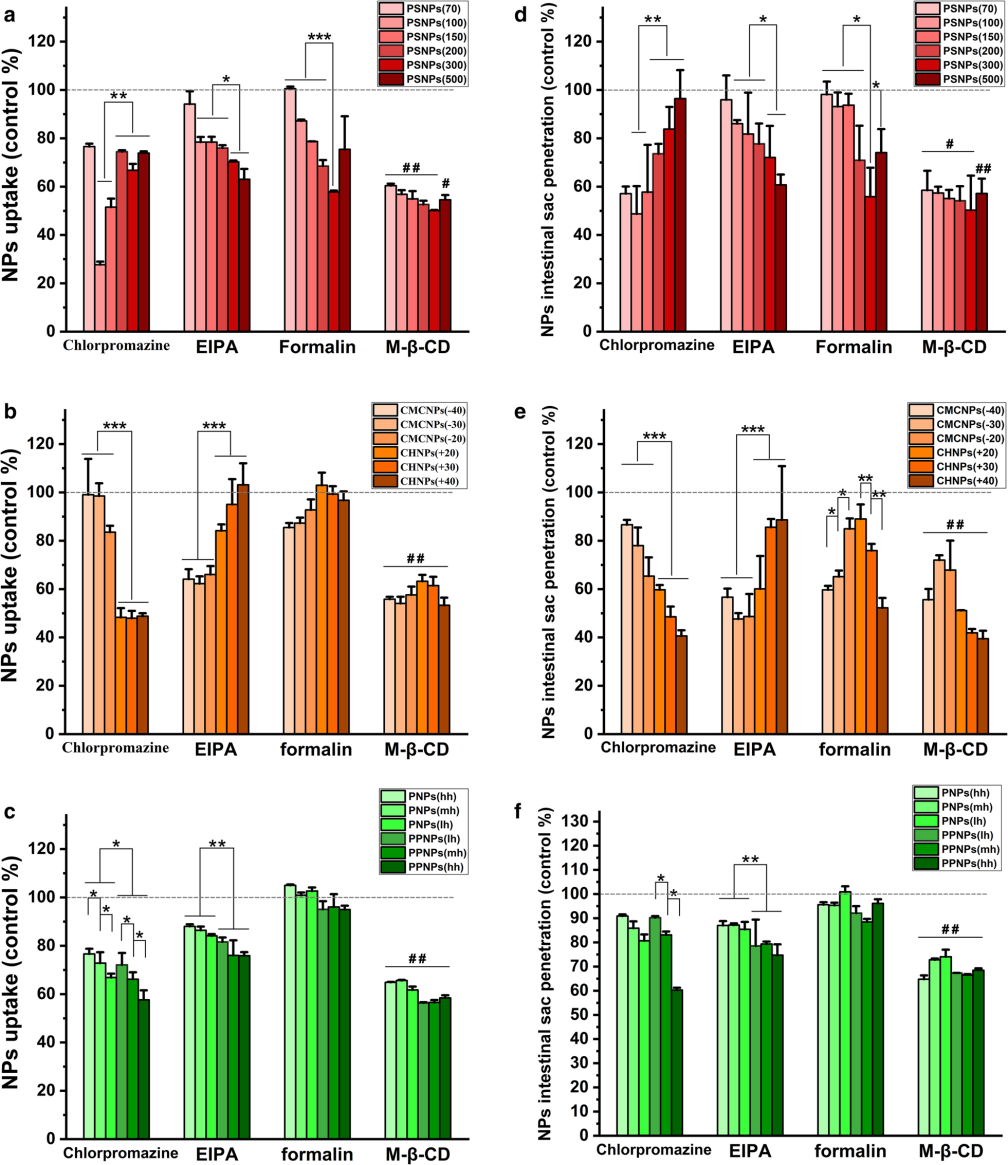
Study on the endocytosis of model NPs: Relative uptake of PSNPs (**a**), CMCNPs, CHNPs (**b**), PNPs and PPNPs (**c**) by caco-2 cells after adding endocytosis inhibitor(data are mean ± SD, n = 3, *p < 0.05,**p < 0.01, ***p < 0.001; Compared with the control group without any inhibitor, ^#^p < 0.05, ^##^p < 0.01). The relative permeability of PSNPs (**d**), CMCNPs, CHNPs (**e**), PNPs and PPNPs (**f**) in the eversion intestinal sac model after adding endocytosis inhibitor (data are mean ± SD, n = 3, *p < 0.05,**p < 0.01, ***p < 0.001; Compared with the control group without any inhibitor, ^#^p < 0.05, ^##^p < 0.01)

To understand the effects of the NPs properties on their uptake, a specific inhibition method was used to study the infiltration mechanisms. Chlorpromazine inhibits clathrin-mediated endocytosis, EIPA inhibits macropinocytosis, formalin is an inhibitor of caveolin-mediated endocytosis, and M-β-CD inhibits lipid raft-mediated endocytosis [47, 48]. These inhibitors were applied to the cell uptake model (Fig. 5a–c) and the rat everted intestinal sac model (Fig. 5d–f), and the effects on cell uptake and intestinal sac permeation were calculated.

Of the PSNPs groups, PSNPs (100) and PSNPs (150) were most affected after clathrin-mediated endocytosis was blocked. After caveolin-mediated endocytosis was blocked, the cellular uptake of PSNPs (300) was reduced the most. After blocking macropinocytosis, NPs uptake showed positive correlation with particle size. All PSNPs showed a reduction in uptake of more than 35% after lipid rafts were inhibited. For the CMCNPs and CHNPs groups, the negatively charged CMCNPs exhibited a greater reduction after macropinocytosis was blocked. The absorption of positively charged CHNPs was clearly affected when clathrin-mediated endocytosis was blocked. The cell uptake of CHNPs (+20), CHNPs (+30), and CHNPs (+40) reduced by 52%, 52%, and 51%, respectively, and the intestinal penetration of CHNPs (+20), CHNPs (+30), and CHNPs (+40) reduced by 40%, 51%, and 59% respectively. An observation that was only clearly made for the animal model was that NPs with lower charge magnitude were less affected when caveolin-mediated endocytosis was blocked. For the PNPs and PPNPs, after clathrin-mediated endocytosis was inhibited, the cellular uptake of PPNPs with longer PEG chains and PNPs made from more hydrophilic PLGA had a greater tendency to be affected. After macropinocytosis was inhibited, the uptake of PPNPs containing PEG components was slightly more affected than that of PNPs. All PNPs and PPNPs were only slightly affected after caveolin-mediated endocytosis was inhibited. Of the various inhibitor treatments, PNPs and PPNPs almost all showed the greatest reduction in uptake when the lipid raft structure was destroyed.

**Fig. 6:**
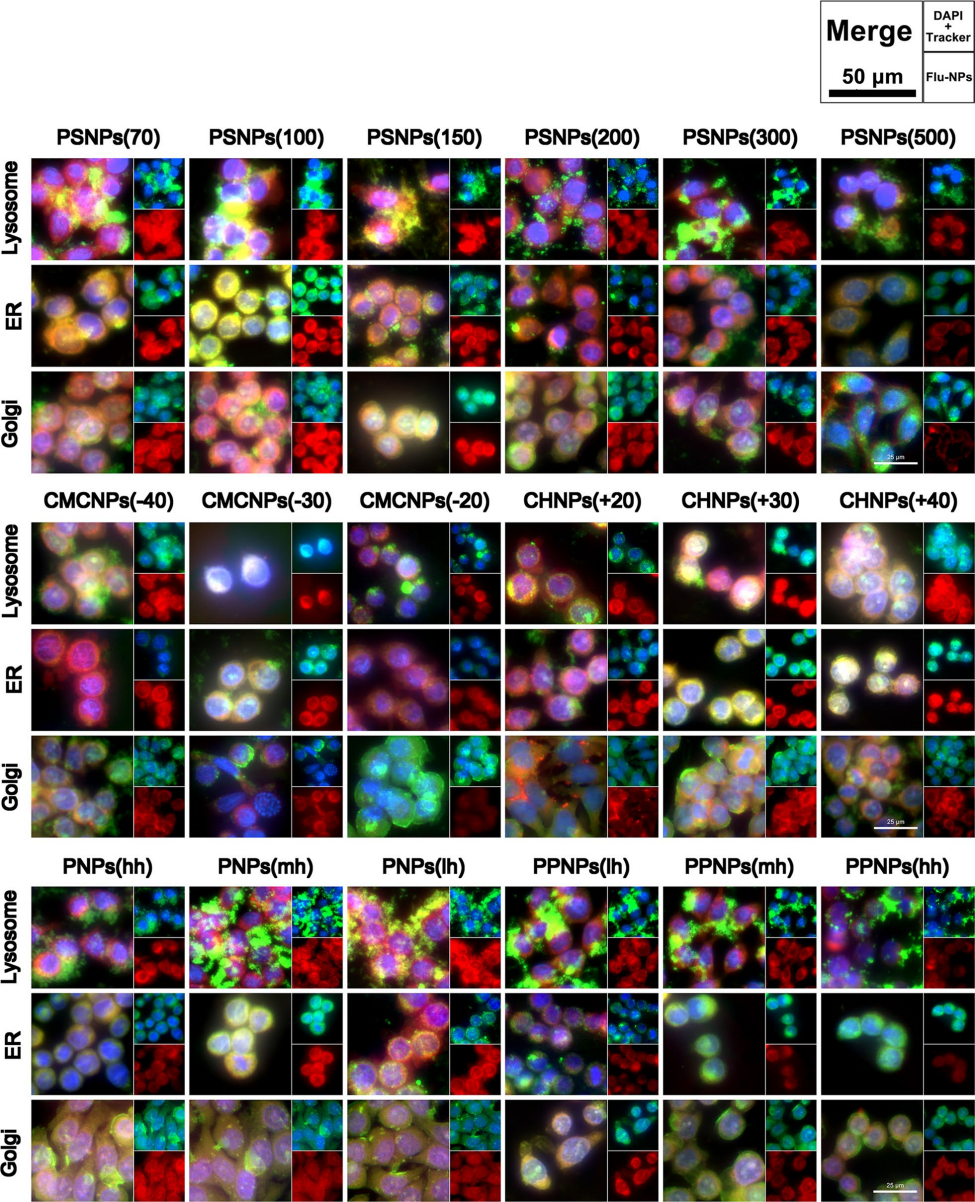
Colocalization images of model NPs with lysosomes, ER and Golgi complexes

To understand the intracellular distribution of the model NPs after entering the cell, Lyso-Tracker, ER-Tracker, and Golgi-Tracker were used to label lysosomes, endoplasmic reticulum, and Golgi, respectively, and imaging was conducted (Fig. 6). All model NPs were labelled with red fluorescent probes, the nuclei were stained blue, and the three cell structures—lysosomes, endoplasmic reticulum, and Golgi apparatus—exhibited green fluorescence.

After entering the intestinal cells, the polymer nanoparticles need to undergo intracellular transport and then exit the cell at the basal side to complete the absorption process. Therefore, the method of specific inhibition was used again to explore the intracellular transport mechanism and exocytosis of model NPs. Bafilomycin A1 is an inhibitor of endosomal acidification. Brefeldin A can prevent the transport of NPs from the ER to the Golgi complex, and monensin can block transport from the Golgi complex to the plasma membrane [49, 50].

**Fig. 7:**
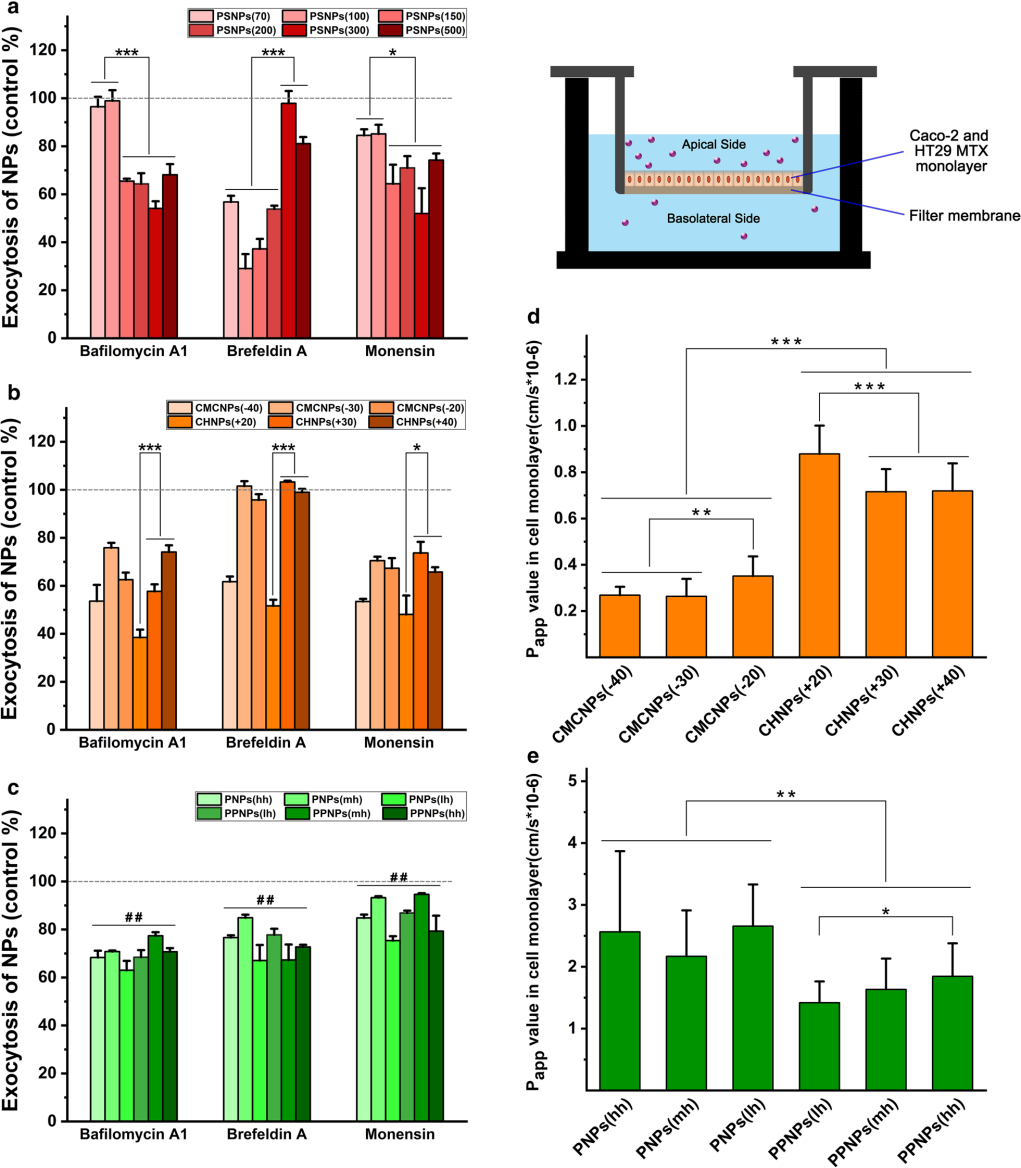
Study on transport and exocytosis of model NPs: Effects of three inhibitors on the exocytosis of caco-2 cells on PSNPs (**a**), CMCNPs, CHNPs (**b**), PNPs and PPNPs (**c**) (data are mean ± SD, n = 3, *p < 0.05, ***p < 0.001; Compared with the control group without any inhibitor, ^##^p < 0.01). P_app_ values of CMCNPs, CHNPs (**d**), PNPs and PPNPs (**e**) permeation across cell monolayers from donor to acceptor compartments (data are mean ± SD, n = 15, *p < 0.05, **p < 0.01, ***p < 0.001)

The results (Fig. 7a–c) show that the exocytosis of all PNPs and PPNPs groups was affected by the three inhibitors. The process of their exocytosis after internalization may involve the reactivation of vesicles and transport to the ER, Golgi complex, and cell membrane in turn. For the PSNPs groups, the exocytosis of PSNPs (100) was more significantly inhibited by bafilomycin A1 and monensin, while the inhibitory effect of brefeldin A on exocytosis was more pronounced for NPs with a particle size less than 300 nm. This indicates that the activation of retransportation after entering the cell occurs more commonly for NPs with a particle size of more than 100 nm, while subsequent transport from the endoplasmic reticulum to the Golgi complex occurs more commonly for NPs with a particle size of less than 300 nm. For the CMCNPs and CHNPs groups, CHNPs (+20) were most significantly inhibited by the three inhibitors. The exocytosis of CHNPs (+20) treated with bafilomycin A1, brefeldin A, and monensin reduced by 62%, 48%, and 52%, respectively. The lower degree of positive charge may have a more positive tendency to exocytosis after entering the cell.

Caco-2 and HT29-MTX monolayer cell models formed in the Transwell were used to evaluate the monolayer cell transport of CMCNPs, CHNPs, PNPs, and PPNPs. The results (Fig. 7d, e) show that the trans-cell transport of positively charged CHNPs was significantly higher than that of negatively charged CMCNPs. The average P_app_ of CHNPs was 2.61 times of that of CMCNPs. When NPs have the same kind of charge, the lower level of positive or negative electric charge gain more transport value. The transport volume of PPNPs containing PEG components was slightly lower than that of PNPs. The average P_app_ of PNPs was 1.51 times of that of PPNPs, and in each group of PPNPs there was a tendency for the P_app_ value to increase as the PEG chain became longer.

## Discussion

Oral administration has always been considered one of the best administration routes, however some poorly soluble small molecule drugs and macromolecular proteins, peptides, and nucleic acid drugs can be limited by degradation or being difficult to absorb in the gastrointestinal tract, making their oral administration impractical. However, there are solutions to these challenges. The enteric coating can protect drugs in the gastric juice, however it only slightly improves the drug absorbance in the intestine. The use of penetration enhancers can enhance the gastrointestinal absorption of drugs [51], but there may be safety issues for long-term administration. The design of oral delivery vehicles for drugs has been extensively studied. Drug carriers can encapsulate therapeutics in carrier materials or special structures to provide a degree of protection [52, 53]. Then, the characteristics of the drug carrier supersede those of the drug itself and become the decisive factor in absorption [54]. Drug carriers with a particle size of more than 5 μm are hardly absorbed in the intestinal tract. In addition, carrier models based on colloidal systems are difficult to stabilize in the gastrointestinal environment. Therefore, the main object of our research was polymer nanoparticles based on solid systems. We studied the effects of their particle size, surface charge, and surface hydrophilicity/hydrophobicity on the intestinal absorption process in depth.

### Particle size

Size is an important parameter for NP drug carriers [55, 56]. A study by Li et al. showed that the intestinal cell transport capacity of NPs is size dependent [40]. Agata et al. found that, following oral delivery, the absorption of smaller NPs was higher than that of larger ones [57]. Khin et al. also conducted research on oral delivery systems based on NPs and found that the smallest particles (50 nm) showed the lowest absorption rate, which indicated that the particle size effect may have a lower limit; outside of which size no longer plays a key role in the degree of absorption [55].

Polystyrene nanoparticles were used as model NPs and were labeled with red fluorescence. When observed by TEM, all PSNPs were approximately spherical with uniform particle sizes of 70, 100, 150, 200, 300, and 500 nm. In deionized water, PBS, and artificial intestinal fluid. The PSNPs maintained stable fluorescence, which fulfilled the requirements for this study.

The intestinal mucus layer is the first major obstacle to the absorption of NPs in the intestine, and this was supported by the experimental results. When the mucus was removed from the small intestine tissue, the absorption of all PSNPs in the small intestine increased. In addition, compared with the control group, the increase in NPs absorption showed a trend of increasing with the increase of particle size, which indicates that larger particle sizes were more hindered by the mucus layer. If the intestinal mucus was removed after incubation with PSNPs (and NPs retained in the mucus were removed at the same time), the PSNPs with larger particle size were relatively less well retained in the intestinal tissue. This also indicates that large NPs are more likely to stay in the mucus layer.

The second major physiological barrier to the absorption process of NPs in the small intestine is the small intestinal epithelial cell barrier. To overcome this obstacle via the transcellular pathway, NPs must enter the small intestinal epithelial cells on the intestinal lumen after penetrating the mucus layer, then be ejected from the cells after passing through the intracellular polar transport to reach the basal side, which completes the absorption process [35]. After co-culturing caco-2 and HT29-MTX cells for 14 days, we performed imaging observation and quantitative measurement of the cellular uptake of PSNPs. PSNPs (100) showed the highest cellular uptake. When the NPs size exceeded 100 nm, the cell uptake showed a negative correlation with the size of the NPs. When the particle size increased to 200 nm, compared with PSNPs (100), the difference of the cellular uptake began to be significant. In the study of the PSNPs endocytosis pathway, chlorpromazine—an inhibitor of clathrin-mediated endocytosis—had the most marked inhibitory effect on PSNPs (100) and PSNPs (150). Formalin—an inhibitor of caveolin-mediated endocytosis—had the most obvious inhibitory effect on PSNPs (300). These results show that, within the scope of this study, clathrin-mediated endocytosis of NPs tends to favor a particle size of 100–150 nm, while caveolin-mediated endocytosis tends to involve NPs with a particle size of approximately 300 nm. After caco-2 cells ingested PSNPs, the NPs were removed from the culture environment and the process of exocytosis was studied. Bafilomycin A1 inhibits the reactivation of vesicles formed after NPs enter cells, and its inhibitory effect on PSNPs with a particle size of more than 100 nm was the most marked. Brefeldin A inhibits the continued transport processes of NPs on the ER, and was found to inhibit the exocytosis of PSNPs with a particle size less than 300 nm most significantly. We found that, within the scope of our research, when the particle size of PSNPs was less than 100 nm, the further transport effect after entering the cell was weakened. However, when the PSNPs size exceeded 300 nm, further intracellular transport from the endoplasmic reticulum was affected.

In terms of overcoming the entire small intestinal absorption barrier, the absorption of PSNPs showed negative correlation with particle size, and small NPs showed higher penetration or absorption values. In the isolated everted intestinal sac model, when the particle size increased to 200 nm, the difference in penetration compared with PSNPs (70) became significant. When the particle size reached 500 nm, the significance level of the difference in penetration became highly significant. In the in vivo intestinal ring absorption model, when the particle size increased to 300 nm, the difference between the absorption values of PSNPs (300) and PSNPs (70) also became significant.

### Surface potential

Surface charge is a non-negligible characteristic in the oral intestinal absorption of NPs [58]. Rieux et al. found that optimizing the particle size of oral delivery systems is important, but that simultaneously optimizing the surface properties (charge, hydrophobicity) is also crucial [11]. The polyphosphate NPs prepared by Akkus et al. penetrated the mucus barrier to a greater extent than dephosphorylated polyphosphate NPs. In addition, if the particulate system was unable to penetrate the mucus barrier well, it was cleared quickly from the mucosa [59]. Akkus believes that this is owing to the negative surface charge of the polyphosphate NPs [60].

Water-soluble chitosan nanoparticles prepared by graft copolymerization were used as the model NPs for the surface potential group, and the NPs were fluorescently labeled with RhB. The particle diameters of all RhB-CMCNPs and RhB-CHNPs determined by DLS measurement were in the range 155 ± 15 nm and had a low PDI. The TEM images showed that the size of the NPs was smaller than that measured by DLS, however the sizes of the CMCNPs and CHNPs in each group were still similar and both appeared to be approximately spherical. In addition, the fluorescent NPs maintained stable fluorescence in deionized water, PBS, and artificial intestinal fluid. Therefore, the prepared CMCNPs and CHNPs met the requirements for the surface potential group for this study.

In the intestinal mucus layer permeability study, positive charge on the surface of the NPs was found to prevent them from passing through the mucus layer and meant that CHNPs were more likely to be retained in the mucus. In addition, as the degree of negative charge on the surface of the CMCNPs increased, their penetration in mucus decreased and their retention increased. The in vivo animal model showed similar results. The positive charge on the surface made CHNPs more likely to remain in the intestinal mucus than CMCNPs, instead of penetrating the mucus layer.

In the intestinal epithelial cell barrier penetration study, different situations emerged. CHNPs generally showed better uptake than CMCNPs. The positive charge on the surface of the NPs was advantageous when they entered the intestinal epithelial cells. In the study of the NPs endocytosis pathway, we found that the most marked difference between CHNPs and CMCNPs was that CHNPs were more affected by chlorpromazine, which indicates that clathrin-mediated endocytosis tends to favor NPs with a positive zeta potential. In the study of CHNPs and CMCNPs transport in monolayer cells, the positively charged CHNPs showed a higher transport capacity than CMCNPs. Compared with the advantages in cell uptake, CHNPs appeared to have a more obvious advantage in the transport results of monolayer cells. The overall cellular uptake of CHNPs was 1.61 times of that of CMCNPs, and the apparent permeability coefficient value on the monolayer cell model was 2.61 times of that of CMCNPs. This may be because the positively charged CHNPs also have the advantage of passing through monolayer cells in ways other than the transcellular pathway. In addition, the transport of CHNPs (+20) in the monolayer cell model was greater than that of the other two groups of CHNPs with higher positive charges. From the findings of the study of its exocytosis process, it can be seen that CHNPs (+20) exhibited the most obvious inhibitory effect of the three intracellular transport inhibitors. Therefore, we speculate that CHNPs (+20) have the highest transport volume. An important factor is that they had a greater tendency to be transported and exit after entering the cell. In terms of overcoming the overall small intestinal absorption barrier, within the scope of our research, negatively charged NPs exhibited significantly higher absorption or adsorption values than positively charged NPs. The average penetration of CMCNPs in the isolated small intestine was 1.38 times of that of CHNPs.

### Surface hydrophobicity/hydrophilicity

The surface pro-hydrophobics of the NPs were also considered in this study. Yuan et al. found that an appropriate hydrophilic modification (such as PEGylation) is important to make NPs that would otherwise be mucosal adhesives, inert during gastrointestinal transport and absorption. The optimal extent of hydrophilic modification of NPs requires follow-up research [61].

(PEG-)PLGA nanoparticles were selected as model NPs for studying the role of hydrophobicity/hydrophilicity in intestinal absorption. DLS measurements and TEM observation showed similar results. The particle sizes of all PNPs and PPNPs were in the range of 185-205 nm, and they had a zeta potential of no more than 5 mV and an approximately spherical shape. In the investigation of hydrophobicity/hydrophilicity, the three PNPs and three PPNPs exhibited the expected differences in hydrophobicity. Two fluorescent reagents (C6 and NR) were encapsulated in the NPs. The fluorescence of the NPs was stable in deionized water, PBS, and artificial intestinal fluid. C6-PNPs, NR-PNPs, C6-PPNPs, and NR-PPNPs were used as model NPs in the study.

The penetration and retention of NPs in isolated intestinal mucus showed that as the hydrophilicity of the NPs increased, the average penetration volume also tended to increase, although the increase was not significant. PPNPs with hydrophilic PEG surfaces had better overall mucus penetration ability than PNPs, and this advantage was significant. In addition, we found that for both PNPs and PPNPs, NPs made using more hydrophilic materials showed higher mucus retention. Increased hydrophobicity made it more difficult for NPs to pass through the mucus, but did not cause greater retention. That is, within a certain range, an increase in the hydrophilicity of the NPs increased the amount of mucus transport and mucus retention. We speculate that the challenge in entering the mucus layer faced by more hydrophobic NPs becomes the main obstacle in the process of transmucosal penetration. Similar conclusions can be drawn for the interaction of NPs with intestinal mucus in the in vivo animal models. The more hydrophilic NPs were less obstructed by the intestinal mucus, and at the same time, there was no reduced mucus retention. NPs was made of more hydrophilic materials and NPs modified with hydrophilic components entered the intestinal mucus more actively and achieved better mucus penetration.

Different results were obtained in the study of the interaction between NPs and intestinal cells. PPNPs modified with hydrophilic components showed less cellular uptake and less monolayer cell transport. However, at the same time, as the length of the PEG chain increased, the monolayer cell transport of PPNPs also tended to increase slightly. All PNPs and PPNPs were significantly inhibited by M-β-CD during endocytosis. This showed that the lipid raft structure plays an important role in the endocytosis of NPs with PLGA as the core. In addition, the more hydrophilic NPs will be slightly more inhibited by chlorpromazine. This indicates that there may be slight selectivity for hydrophilic NPs in clathrin-mediated endocytosis. At the same time, modification with PEG also affected the cellular uptake of NPs after EIPA blocked the effect of macropinocytosis. After all groups of PNPs and PPNPs entered the cell, the process of exocytosis was significantly affected by the three transport inhibitors, which indicates that the activation of vesicles, the transfer of endoplasmic reticulum, and the Golgi complex are all important factors in their intracellular transport.

In the study of the penetration and absorption of intact small intestine tissue, PPNPs showed higher penetration or absorption in the in vitro and in vivo rat small intestine. In addition, within the scope of our research, PPNPs with longer PEG chains exhibited stronger small intestinal absorption and adsorption.

## Conclusion

In this study, we successfully prepared model nanoparticles with single variable differences in particle size, surface charge, and surface hydrophilicity/hydrophobicity, and then studied the role of these basic properties in the oral intestinal absorption of the polymer nanoparticles. We conducted in-depth research by separating the two main stages of intestinal absorption. We found that the absorption of NPs showed a tendency to increase with increasing particle size. The mucus layer hindered larger particles making them more likely to be retained in the layer. In addition, positive surface charge prevented NPs from passing through the mucus layer. Increasing the magnitude of the negative surface charge of NPs also reduced their permeation in mucus and increased their retention. Overall, PPNPs with a hydrophilic PEG surface penetrated mucus better than PNPs. In addition, as their hydrophobicity increased, PNPs and PPNPs found it more difficult to pass through mucus, but were not better retained. PSNPs with a particle size of 100 nm showed the highest intestinal cellular uptake. When the size of the NPs exceeded 100 nm, cellular uptake was negatively correlated with the size of the NPs. When the particle size was increased to 200 nm, the difference in cellular uptake compared with PSNPs (100) started to become significant. Within the scope of our research, it was found that a particle size of 100–150 nm was conducive to clathrin-mediated endocytosis of NPs. Caveolin-mediated endocytosis tended to be observed for NPs with a particle size of around 300 nm. When the particle size of PSNPs was less than 100 nm, the effect of further transport after entering the cell was less pronounced. However, when the size of the PSNPs exceeded 300 nm, it further affected the intracellular transport of the endoplasmic reticulum. The positively charged CHNPs showed better intestinal cell absorption and monolayer cell transport than the negatively charged CMCNPs. The overall cellular uptake of CHNPs was 1.61 times of that of CMCNPs, and the apparent permeability coefficient value on the monolayer cell model was 2.61 times that of CMCNPs. The difference in the endocytosis pathway was reflected in the clathrin-mediated endocytosis, and it was observed that NPs with a positive zeta potential tended to be taken up by clathrin-mediated endocytosis. In addition, CHNPs (+20) had a greater tendency to migrate and exit after entering the cell than the other positively charged particles. PPNPs modified with hydrophilic components showed less cellular uptake and less monolayer cell transport. However, as the length of the PEG chain increased, the monolayer cell transport of PPNPs also tended to increase gradually. In clathrin-mediated endocytosis, hydrophilic NPs showed slight selectivity. Modification with PEG also affected the cellular uptake of NPs through macropinocytosis. The activation of vesicles, endoplasmic reticulum transport, and the Golgi apparatus were all important factors in the intracellular transport of PNPs and PPNPs.

In summary, smaller size, low magnitude negative charge, and moderate hydrophilicity can help NPs pass through the small intestinal mucus layer more easily. Then the proper size, positive surface charge, and hydrophobic properties help NPs complete the process of transintestinal epithelial cell transport. NPs with different characteristics show different behavioral trends in the process of overcoming the absorption barrier of the small intestine. Reliable oral administration of difficult-to-absorb drugs is still an important topic in the field of formulations. We believe that the future challenge will no longer be how to protect drugs but how to adapt the characteristics of the drug delivery system to the needs of intestinal absorption. To deepen our understanding of intestinal cells ingesting particles, more effort must be made to conduct in-depth studies in the future.

## Abbreviations

NPs: Nanoparticles
FDA: Food and Drug Administration
PSNPs: Polystyrene nanoparticles
CMCNPs: Carboxymethyl chitosan nanoparticles
CHNPs: Chitosan hydrochloride nanoparticles
RhB: Rhodamine B
PLGA: Poly(lactic-co-glycolic acid)
PEG: Polyethylene glyco
C6: Coumarin 6
NR: Nile red
Flu-PSNPs: Fluorescent polystyrene nanoparticles
M-β-CD: Methyl-β-cyclodextrin
EIPA: 5-(*N*-ethyl-*N*-isopropyl) amiloride
DAPI: 4,6-Diamino-2-phenyl indole
Lyso: Lysosome
ER: Endoplasmic reticulum
Golgi: Golgi complex
SD: Sprague–Dawley
MMA: Methyl methacrylate
APS: Ammonium persulfate
EDC·HCl: 1-Ethyl-3-(3-dimethylaminopropyl)carbodiimide hydrochloride
NHS: *N*-Hydroxysuccinimide
PVA: Polyvinyl alcohol
DLS: Dynamic light scattering
PDI: Polydispersity index
SEM: Scanning electron microscope
TEM: Transmission electron microscope
PBS: Buffered saline
DMEM: Dulbecco’s modified eagle media
TEER: Transepithelial resistance
HBSS: Hank’s balanced salt solution
NAC: *N*-acetyl-l-cysteine
